# Method for the synthesis of flavonoid nitrogen mustard derivatives

**DOI:** 10.1016/j.mex.2020.100903

**Published:** 2020-04-25

**Authors:** Jinglei Song, Meixuan Yu, Xi Yan, Haijun Hao

**Affiliations:** a College of Chemistry, Beijing Normal University, Beijing, 100875, PR China; b Department of Organic Chemistry, College of Science, Beijing University of Chemical Technology, Beijing, 100029, PR China

**Keywords:** Chlorination, Organic synthesis, Yield

## Abstract

We have prepared a kind of new type of flavonoid nitrogen mustard derivatives with broad-spectrum antitumor activity, which have not been reported in the literature. In the process of preparing intermediate (the flavonoid diethanolamine derivatives) of the target compound, we found that the reasonable separation method is silica gel column chromatography with eluent (MeOH/CH_2_Cl_2_, 1:40). The experimental results show that the composition of eluent is an important factor to get high yield of the intermediate, which will directly affect the final yield of the target compound. Acetonitrile is the suitable solvent in the reaction system, and the optimized reaction condition is reaction under reflux condition for 48 hours. Several new flavonoid nitrogen mustard derivatives were synthesized with high yield using the above method.

• A method for the synthesis of flavonoid nitrogen mustard derivatives was introduced.
• The reasonable purification conditions and the optimized reaction time were recommended.

## Graphical abstract

**Figure:**
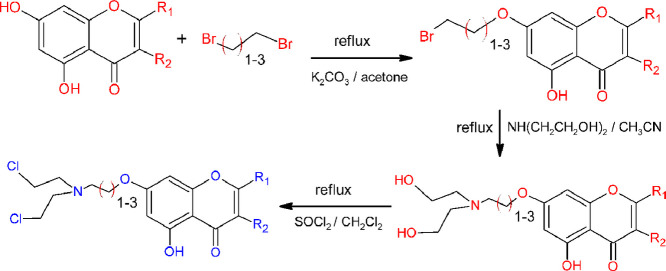

**Table:** Specifications Table

Subject Area:	Chemistry
More specific subject area:	*Medicinal chemistry*
Method name:	*Effect of eluent and solvent on yield in the reaction system*
Name and reference of original method:	*If applicable, include full bibliographic details of the main reference(s) describing the original method from which the new method was derived.*
Resource availability:	*If applicable, include links to resources necessary to reproduce the method (e.g. data, software, hardware, reagent)*

## Method details

### Instruments required

**Scheme 1:**
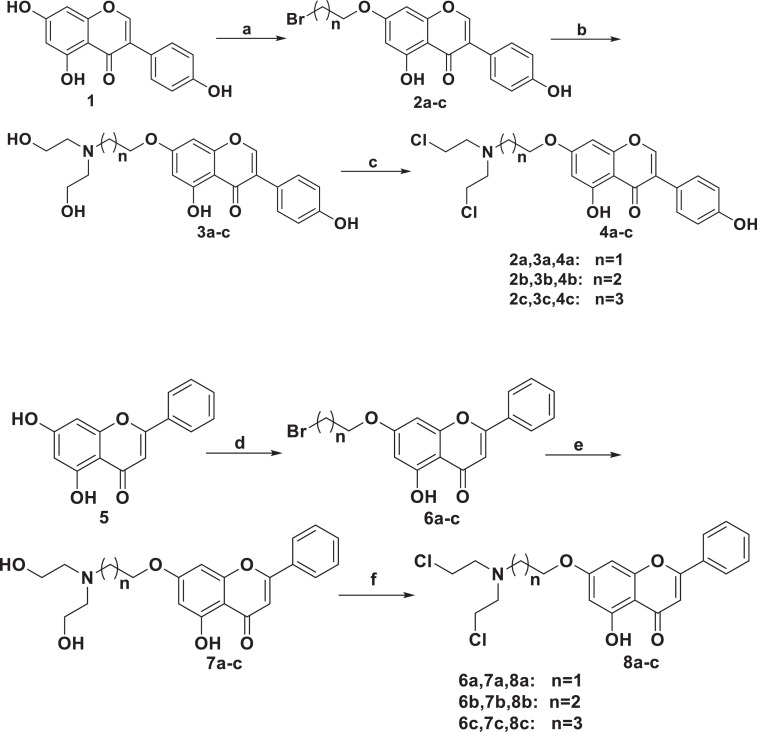
Synthesis of genistein and chrysin nitrogen mustard derivatives ^[^^1^^]^ (4a-c, 8a-c). Reagents and conditions: (a) K_2_CO_3_, acetone, 1,2-dibromoethane, 1,3-dibromopropane, or 1,4-dibromobutane, reflux, 8h, 69%-75%(for 2a-c); (b) NH(CH_2_CH_2_OH)_2_, CH_3_CN, reflux, 48h, 85%-88%(for 3a-c); (c) SOCl_2_, CH_2_Cl_2_, reflux, 48h, 87%-92%(for 4a-c); (d) K_2_CO_3_, acetone, 1,2-dibromoethane, 1,3-dibromopropane, or 1,4-dibromobutane, reflux, 8h, 67%-89%(for 6a-c); (e) NH(CH_2_CH_2_OH)_2_, CH_3_CN, reflux, 48h, 89%-96%(for 7a-c); (f) SOCl_2_, CH_2_Cl_2_, reflux, 48h, 79%-84%(for 8a-c).

Rotary evaporator, filter flask, Busher funnel, glassware (round bottom flask, beakers, condenser, chromatographic column and watch glass), silica-gel thin layer chromatography plates, chromatographic column, Electric heating jacket with temperature controller, Barometer, Portable UV detection lamp, Melting point instrument, Nuclear magnetic resonance instrument, Mass spectrometer. Scheme 1.

### Procedure

To a solution of genistein (1mmol, 1eq) in 50 mL anhydrous acetone was added potassium carbonate (2mmol, 2eq) and the mixture was stirred for 15min at room temperature. Then 1, 2-dibromoethane, 1, 3-dibromopropane, or 1, 4-dibromobutane (10 mmol, 10eq) was slowly added. The reaction was heated to reflux for 8h. The solvent was removed by evaporation under reduced pressure. The product was washed with 5 mL (× 3) petroleum ether and then 10 mL (× 3) distilled water in succession, and filtered under reduced pressure. Then the residue was dried and purified by silica gel column chromatography (MeOH/CH_2_Cl_2_, 1:60) to give compounds 2a-c [[1], [2], [3], [4],^7^].

To a solution of 2a-c (1mmol, 1eq) in 50 mL acetonitrile was added diethanolamine (10mmol, 10eq). The reaction was heated to reflux for 48h at 85°C. The mixture was cooled to room temperature to crystallize, filtered under reduced pressure and washed with water. Then the residue was dried and purified by silica gel column chromatography (MeOH/CH_2_Cl_2_, 1:40) to give compounds 3a-c.

To a solution of 3a-c (1mmol, 1eq) in 50 mL CH_2_Cl_2_ was added SOCl_2_ (8mmol, 8eq). The reaction was heated to reflux for 48h. The solvent was removed by **rotary evaporator**. Then the residue was dried and purified by silica gel column chromatography (MeOH/CH_2_Cl_2_, 1:60) to give compounds 4a-c [1,5,6].

To a solution of chrysin (1mmol, 1eq) in 50 mL anhydrous acetone was added potassium carbonate (4mmol, 4eq) and the mixture was stirred for 15min at room temperature. Then 1,2-dibromoethane, 1,3-dibromopropane, or 1,4-dibromobutane (10mmol, 10eq) was slowly added. The reaction was heated to reflux for 8h. The solvent was removed under vacuum. The resulting residue was washed with petroleum ether and distilled water successively, and filtered under reduced pressure. Then the residue was dried and purified by silica gel column chromatography (CH_2_Cl_2_) to give compounds 6a-c.

To a solution of 6a-c (1mmol, 1eq) in 50mL acetonitrile was added diethanolamine (10mmol, 10eq). The reaction was heated to reflux for 48h. The mixture was cooled to room temperature to crystallize, filtered under reduced pressure and washed with water. Then the residue was dried and purified by silica gel column chromatography (MeOH/CH_2_Cl_2_, 1:60) to give compounds 7a-c.

To a solution of 7a-c (1mmol, 1eq) in 50mL CH_2_Cl_2_ was added SOCl_2_ (8mmol, 8eq). The reaction was heated to reflux for 48h. The solvent was removed by **rotary evaporator**. Then the residue was dried and purified by silica gel column chromatography (MeOH/CH_2_Cl_2_, 1:100) to give compounds 8a-c [1,5,6].

## Appendix Supplementary materials

Image, application 1
