# Supplementary material for: Method for the synthesis of flavonoid nitrogen mustard derivatives
Source: MethodsX. 2020 Apr 25;7:100903. doi: 10.1016/j.mex.2020.100903 (PMC7210452; doi:10.1016/j.mex.2020.100903)
Supplement: Supplementary file 1 [file mmc1.docx]

**Supplementary material *and/or* Additional information:**

7-(2-bromoethoxy)-5-hydroxy-3-(4-hydroxyphenyl)-4H-chromen-4-one (2a)

Yield 70%, yellow solid. mp 181-182^0^C; ^1^H NMR (400 MHz, DMSO-D6) δ 12.96 (s, 1H), 9.58 (s, 1H), 8.41 (s, 1H), 7.38 (d, *J* = 8.6 Hz, 2H), 6.82 (d, *J* = 8.7 Hz, 2H), 6.69 (d, *J* = 2.3 Hz, 1H), 6.43 (d, *J* = 2.3 Hz, 1H), 4.47 – 4.43 (m, 2H), 3.86 – 3.78 (m, 2H).

7-(3-​bromopropoxy)-5-hydroxy-3-(4-hydroxyphenyl)-4H-chromen-4-one (2b)

Yield 69%, yellow solid. mp 130-132^0^C; ^1^H NMR (600 MHz, DMSO-D6) δ 9.59 (s, 1H), 8.41 (s, 1H), 7.38 (d, *J* = 8.6 Hz, 2H), 6.81 (d, *J* = 8.6 Hz, 2H), 6.68 (d, *J* = 2.2 Hz, 1H), 6.42 (d, *J* = 2.2 Hz, 1H), 4.20 (t, *J* = 6.0 Hz, 2H), 3.66 (t, *J* = 6.6 Hz, 2H), 2.26 (p, *J* = 6.3 Hz, 2H).

7-(4-​bromobutoxy)-5-hydroxy-3-(4-hydroxyphenyl)-4H-chromen-4-one (2c)

Yield 75%, yellow solid. mp 138-140^0^C; ^1^H NMR (400 MHz, DMSO-D6) δ 12.95 (s, 1H), 9.59 (s, 1H), 8.41 (s, 1H), 7.39 (d, *J* = 8.7 Hz, 2H), 6.82 (d, *J* = 8.7 Hz, 2H), 6.66 (d, *J* = 2.3 Hz, 1H), 6.41 (d, *J* = 2.3 Hz, 1H), 4.14 (t, *J* = 6.3 Hz, 2H), 3.62 (t, *J* = 6.6 Hz, 2H), 2.01 – 1.92 (m, 2H), 1.90 – 1.81 (m, 2H).

7-​[2-​[bis(2-​hydroxyethyl)​amino]​ethoxy]​-​5-​hydroxy-​3-​(4-​hydroxyphenyl)​-4H-chromen-​4-​one (3a)

Yield 88%, light yellow solid. mp 139-141^0^C; ^1^H NMR (400 MHz, METHANOL-D4) δ 8.08 (s, 1H), 7.37 (d, *J* = 8.5 Hz, 2H), 6.84 (d, *J* = 8.5 Hz, 2H), 6.51 (d, *J* = 1.9 Hz, 1H), 6.35 (d, *J* = 2.0 Hz, 1H), 4.15 (t, *J* = 5.6 Hz, 2H), 3.63 (t, *J* = 5.7 Hz, 4H), 3.01 (t, *J* = 5.6 Hz, 2H), 2.77 (t, *J* = 5.7 Hz, 4H). MS (ESI): Calcd.C_21_H_23_NO_7_, [M+H^+^] m/z: 402.15, found: 402.1180.

7-​[3-​[bis(2-​hydroxyethyl)​amino]​propoxy]​-​5-​hydroxy-​3-​(4-​hydroxyphenyl)​-4H-chromen-​4-​one (3b)

Yield 85%, light yellow solid. mp 157-159^0^C; ^1^H NMR (600 MHz, METHANOL-D4) δ 8.09 (s, 1H), 7.38 (d, *J* = 8.4 Hz, 2H), 6.85 (d, *J* = 8.5 Hz, 2H), 6.52 (s, 1H), 6.35 (s, 1H), 4.14 (t, *J* = 6.0 Hz, 2H), 3.62 (t, *J* = 5.7 Hz, 4H), 2.74 (t, *J* = 7.0 Hz, 2H), 2.68 (t, *J* = 5.7 Hz, 4H), 2.02 – 1.90 (m, 2H). MS (ESI): Calcd.C_22_H_25_NO_7_, [M+H^+^] m/z: 416.16, found: 416.0802.

7-​[4-​[bis(2-​hydroxyethyl)​amino]​butoxy]​-​5-​hydroxy-​3-​(4-​hydroxyphenyl)​-4H-​chromen-​4-​one (3c)

Yield 86%, light yellow solid. mp 141-142^0^C; ^1^H NMR (400 MHz, METHANOL-D4) δ 8.08 (s, 1H), 7.40 – 7.35 (m, 2H), 6.87 – 6.82 (m, 2H), 6.50 (d, *J* = 2.4 Hz, 1H), 6.34 (d, *J* = 2.0 Hz, 1H), 4.07 (t, *J* = 6.4 Hz, 2H), 3.62 (t, *J* = 6.0 Hz, 4H), 2.70 – 2.60 (m, 6H), 1.86 – 1.77 (m, 2H), 1.71 – 1.62 (m, 2H). MS (ESI): Calcd.C_23_H_27_NO_7_, [M+H^+^] m/z: 430.18, found: 430.3159.

7-​[2-​[bis(2-chloroethyl)​amino]​ethoxy]​-​5-​hydroxy-​3-​(4-​hydroxyphenyl)​-4H-chromen-​4-​one (4a)

Yield 92%, light yellow solid. mp 148-150^0^C; ^1^H NMR (600 MHz, METHANOL-D4) δ 8.20 (s, 1H), 7.44 (d, *J* = 8.7 Hz, 2H), 6.90 (d, *J* = 8.7 Hz, 2H), 6.68 (d, *J* = 2.3 Hz, 1H), 6.51 (d, *J* = 2.3 Hz, 1H), 4.60 – 4.54 (m, 2H), 4.11 (t, *J* = 6.2 Hz, 4H), 3.94 – 3.89 (m, 2H), 3.86 (t, *J* = 6.2 Hz, 4H). MS (ESI): Calcd.C_21_H_21_Cl_2_NO_5_, [M+H^+^] m/z: 438.08, found: 438.0252.^13^C NMR (101 MHz, METHANOL-D4) δ 180.45, 163.19, 161.76, 157.63, 157.36, 154.50, 130.11, 122.61, 120.89, 115.16, 105.84, 98.56, 93.12, 63.38, 54.12, 51.43, 37.60.

7-​[3-​[bis(2-chloroethyl)​amino]​propoxy]​-​5-​hydroxy-​3-​(4-​hydroxyphenyl)​-4H-chromen-​4-​one (4b)

Yield 91%, light gray solid. mp 282-289^0^C; ^1^H NMR (600 MHz, METHANOL-D4) δ 8.15 (s, 1H), 7.41 (d, *J* = 8.6 Hz, 2H), 6.88 (d, *J* = 8.6 Hz, 2H), 6.58 (d, *J* = 2.1 Hz, 1H), 6.41 (d, *J* = 2.2 Hz, 1H), 4.24 (t, *J* = 5.6 Hz, 2H), 4.07 (t, *J* = 6.1 Hz, 4H), 3.78 (t, *J* = 6.1 Hz, 4H), 3.65 – 3.55 (m, 2H), 2.36 – 2.30 (m, 2H). MS (ESI): Calcd.C_22_H_23_Cl_2_NO_5_, [M+H^+^] m/z: 452.10, found: 452.0548.^13^C NMR (101 MHz, DMSO-D6) δ 180.92, 164.62, 162.27, 158.10, 157.97, 154.93, 130.64, 123.07, 121.46, 115.65, 106.05, 98.93, 93.38, 66.27, 53.76, 50.44, 37.82, 23.25.

7-​[4-​[bis(2-chloroethyl)​amino]​butoxy]​-​5-​hydroxy-​3-​(4-​hydroxyphenyl)​-4H-​chromen-​4-​one (4c)

Yield 87%, light gray solid. mp 235-250^0^C; ^1^H NMR (600 MHz, METHANOL-D4) δ 8.12 (s, 1H), 7.46 – 7.27 (m, 2H), 6.91 – 6.76 (m, 2H), 6.55 (d, *J* = 2.2 Hz, 1H), 6.38 (d, *J* = 2.2 Hz, 1H), 4.16 (t, *J* = 5.8 Hz, 2H), 4.01 (t, *J* = 6.1 Hz, 4H), 3.72 (t, *J* = 6.0 Hz, 4H), 3.53 – 3.34 (m, 2H), 2.02 – 1.95 (m, 2H), 1.95 – 1.89 (m, 2H). MS (ESI): Calcd.C_23_H_25_ Cl_2_NO_5_, [M+H^+^] m/z: 466.11, found: 466.25.^13^C NMR (101 MHz, DMSO-D6) δ 180.90, 164.91, 162.26, 158.09, 157.98, 154.89, 130.64, 123.02, 121.48, 115.64, 105.92, 98.89, 93.36, 68.36, 53.59, 52.80, 37.80, 25.97, 20.24.

7-​(2-​bromoethoxy)​-​5-​hydroxy-​2-​phenyl-4H-​chromen-​4-​one (6a)

Yield 67%, yellow solid. mp 157-159^0^C; ^1^H NMR (600 MHz, CHLOROFORM-D) δ 12.74 (s, 1H), 7.91 – 7.86 (m, 2H), 7.63 – 7.45 (m, 3H), 6.68 (s, 1H), 6.53 (d, *J* = 2.2 Hz, 1H), 6.38 (d, *J* = 2.2 Hz, 1H), 4.37 (t, *J* = 6.3 Hz, 2H), 3.67 (t, *J* = 6.3 Hz, 2H).

7-(3-​bromopropoxy)-5-hydroxy-​2-​phenyl-4H-​chromen-​4-​one (6b)

Yield 77%, yellow solid. mp 163-167^0^C; ^1^H NMR (400 MHz, CHLOROFORM-D) δ 12.72 (s, 1H), 7.94 – 7.84 (m, 2H), 7.60 – 7.47 (m, 3H), 6.67 (s, 1H), 6.52 (d, *J* = 2.3 Hz, 1H), 6.38 (d, *J* = 2.2 Hz, 1H), 4.20 (t, *J* = 5.8 Hz, 2H), 3.61 (t, *J* = 6.4 Hz, 2H), 2.41 – 2.29 (m, 2H).

7-(4-​bromobutoxy)-5-hydroxy-2-phenyl-4H-chromen-4-one (6c)

Yield 89%, yellow solid. mp 147-148^0^C; ^1^H NMR (400 MHz, CHLOROFORM-D) δ 12.69 (s, 1H), 7.89 – 7.85 (m, 2H), 7.57 – 7.48 (m, 3H), 6.65 (s, 1H), 6.48 (d, *J* = 2.2 Hz, 1H), 6.35 (d, *J* = 2.2 Hz, 1H), 4.07 (t, *J* = 6.0 Hz, 2H), 3.49 (t, *J* = 6.4 Hz, 2H), 2.12 – 2.03 (m, 2H), 2.03 – 1.94 (m, 2H).

7-​[2-​[bis(2-​hydroxyethyl)​amino]​ethoxy]​-​5-​hydroxy-​2-phenyl-4H-chromen-4-one (7a)

Yield 91%, light yellow solid. mp 165-167^0^C; ^1^H NMR (400 MHz, CHLOROFORM-D) δ 12.71 (s, 1H), 7.87 (dd, *J* = 8.0, 1.6 Hz, 2H), 7.58 – 7.47 (m, 3H), 6.65 (s, 1H), 6.51 (d, *J* = 2.2 Hz, 1H), 6.36 (d, *J* = 2.2 Hz, 1H), 4.12 (t, *J* = 5.3 Hz, 2H), 3.66 (t, *J* = 5.2 Hz, 4H), 3.04 (t, *J* = 5.3 Hz, 2H), 2.82 (t, *J* = 5.2 Hz, 4H). MS (ESI): Calcd.C_21_H_23_NO_6_, [M+H^+^] m/z: 386.15, found: 386.0789.

7-[3-[bis(2-hydroxyethyl)amino]propoxy]-​5-​hydroxy-​2-phenyl-4H-chromen-4-one (7b)

Yield 89%, light yellow solid. mp 100-102^0^C; ^1^H NMR (600 MHz, CHLOROFORM-D) δ 12.71 (s, 1H), 7.88 (dd, *J* = 8.1, 1.4 Hz, 2H), 7.58 – 7.48 (m, 3H), 6.66 (s, 1H), 6.51 (d, *J* = 2.2 Hz, 1H), 6.37 (d, *J* = 2.2 Hz, 1H), 4.14 (t, *J* = 5.9 Hz, 2H), 3.66 (t, *J* = 5.2 Hz, 4H), 2.78 (t, *J* = 6.8 Hz, 2H), 2.71 (t, *J* = 5.2 Hz, 4H), 2.07 – 1.88 (m, 2H). MS (ESI): Calcd.C_22_H_25_NO_6_, [M+H^+^] m/z: 400.17, found: 400.1818.

7-[4-[bis(2-hydroxyethyl)amino]butoxy]​-​5-​hydroxy-​2-phenyl-4H-chromen-4-one (7c)

Yield 96%, light yellow solid. mp 83-85^0^C; ^1^H NMR (600 MHz, METHANOL-D4) δ 7.95 (d, *J* = 7.3 Hz, 2H), 7.58 – 7.49 (m, 3H), 6.71 (s, 1H), 6.61 (s, 1H), 6.30 (d, *J* = 1.3 Hz, 1H), 4.06 (t, *J* = 5.9 Hz, 2H), 3.60 (t, *J* = 5.9 Hz, 4H), 2.65 (t, *J* = 5.9 Hz, 4H), 2.63 – 2.59 (m, 2H), 1.83 – 1.77 (m, 2H), 1.69 – 1.62 (m, 2H). MS (ESI): Calcd.C_23_H_27_NO_6_, [M+H^+^] m/z: 414.18, found: 414.1972.

7-​[2-​[bis(2-chloroethyl)​amino]​ethoxy]​-​5-​hydroxy-​2-phenyl-4H-chromen-4-one (8a)

Yield 84%, light yellow solid. mp 138-140^0^C; ^1^H NMR (400 MHz, METHANOL-D4) δ 8.02 (d, *J* = 6.6 Hz, 2H), 7.66 – 7.52 (m, 3H), 6.86 – 6.79 (m, 2H), 6.49 (d, *J* = 1.7 Hz, 1H), 4.61 – 4.53 (m, 2H), 4.08 (t, *J* = 6.0 Hz, 4H), 3.95 – 3.79 (m, 6H). MS (ESI): Calcd.C_21_H_21_Cl_2_NO_4_, [M+H^+^] m/z: 422.08, found: 422.0545.^13^C NMR (101 MHz, DMSO-D6) δ 182.66, 164.12, 164.00, 161.71, 157.79, 132.76, 131.06, 129.71, 127.00, 105.97, 105.83, 99.18, 94.07, 55.47, 54.83, 52.01.

7-​[3-​[bis(2-chloroethyl)​amino]​propoxy]​-​5-​hydroxy-​2-phenyl-4H-chromen-4-one (8b)

Yield 80%, light yellow solid. mp 119-121^0^C; ^1^H NMR (600 MHz, METHANOL-D4) δ 8.02 (d, *J* = 7.1 Hz, 2H), 7.66 – 7.53 (m, 3H), 6.81 (s, 1H), 6.74 (d, *J* = 1.9 Hz, 1H), 6.42 (d, *J* = 2.1 Hz, 1H), 4.28 (t, *J* = 5.6 Hz, 2H), 4.08 (t, *J* = 6.1 Hz, 4H), 3.80 (t, *J* = 6.1 Hz, 4H), 3.67 – 3.58 (m, 2H), 2.40 – 2.33 (m, 2H). MS (ESI): Calcd.C_22_H_23_ Cl_2_NO_4_, [M+H^+^] m/z: 436.10, found: 436.33.^13^C NMR (101 MHz, DMSO-D6) δ 182.55, 164.74, 163.98, 161.69, 157.80, 132.68, 131.06, 129.66, 126.94, 105.87, 105.54, 99.02, 93.81, 66.26, 53.75, 50.41, 37.91, 23.35.

7-​[4-[bis(2-chloroethyl)amino]butoxy]​-​5-​hydroxy-​2-phenyl-4H-chromen-4-one (8c)

Yield 79%, light gray solid. mp 150-151^0^C; ^1^H NMR (600 MHz, METHANOL-D4) δ 8.02 (s, 2H), 7.80 – 7.29 (m, 3H), 6.79 (s, 2H), 6.39 (s, 1H), 4.22 (s, 2H), 4.04 (s, 4H), 3.73 (s, 4H), 3.45 (s, 2H), 2.29 – 1.78 (m, 4H).MS (ESI): Calcd.C_23_H_25_Cl_2_NO_4_, [M+H^+^] m/z: 450.12, found: 450.22.^13^C NMR (101 MHz, DMSO-D6) δ 182.55, 165.08, 163.96, 161.69, 157.84, 132.71, 131.10, 129.71, 126.98, 105.89, 105.43, 99.03, 93.80, 68.46, 53.57, 52.93, 37.66, 26.08, 20.26.
